# Impact of Internet Usage on Depression Among Older Adults: Comprehensive Study

**DOI:** 10.2196/65399

**Published:** 2025-01-31

**Authors:** Lin Guo, Yunwei Li, Kai Cheng, Ying Zhao, Wenqiang Yin, Ying Liu

**Affiliations:** 1 School of Management Shandong Second Medical University Weifang China; 2 School of Humanities and Management Zhejiang Chinese Medical University Hangzhou China

**Keywords:** internet usage, depression, older people, mechanism, heterogeneity, mobile phone

## Abstract

**Background:**

Depression is a widespread mental health issue affecting older adults globally, with substantial implications for their well-being. Although digital interventions have proven effective in high-income countries, research on the potential of internet usage to alleviate depression among older adults in high-income countries remains limited.

**Objective:**

This study aimed to examine the impact of internet usage on depression among older adults in high-income countries by developing a comprehensive theoretical framework and testing key hypotheses.

**Methods:**

Using data from the China Health and Retirement Longitudinal Study (CHARLS), a 2-stage instrumental variable approach was applied to address endogeneity and estimate causal relationships between internet usage and depression.

**Results:**

The findings indicate that internet usage results in a 1.41% reduction in depression levels among older adults. This effect is mediated by four primary mechanisms: (1) enhanced social interaction, (2) increased physical activity, (3) improved intergenerational contact, and (4) expanded access to educational opportunities. A heterogeneity analysis revealed that these effects are more pronounced in urban areas, eastern regions, and regions with superior internet infrastructure.

**Conclusions:**

Internet usage plays a crucial role in alleviating depression among older adults in high-income countries, with regional variations. The findings highlight the need for targeted policy interventions to improve internet access and digital literacy, which can mitigate depression and enhance the mental health of older adults.

## Introduction

### Background

Depression is a prevalent psychological and mental health condition. Globally, the prevalence of depression among the older population aged 60 years and above is 5.7%. This issue is even more pronounced in low- and middle-income countries [1]. Data from the United Nations Economic and Social Commission for Asia and the Pacific indicate that the older population in Southeast Asia is growing rapidly. By 2030, individuals aged 65 years and older are projected to make up over 11% of the total population in the region. Mental health issues among this demographic, particularly depression, are becoming a growing concern. A study by the Asian Mental Health Association shows that depression is particularly prevalent among older adults in Southeast Asia. This is especially true for older adults affected by loneliness, physical health issues, and lifestyle changes, which can contribute to higher rates of depression. Common symptoms of depression in older adults include feelings of isolation, sleep disturbances, and low mood [2].

In China, the prevalence of depression among older adults is estimated to be as high as 25.55% [3]. From 2020 to 2050, China’s older population aged 60 years and older is projected to increase from 263 million to 522 million, with its proportion of the total population rising from 18.7% to 39.5% [4]. Depression significantly affects the quality of life, daily activities, incidence of geriatric diseases, and suicide rates among older adults [5,6]. It not only causes severe harm to the physical and mental health of older adults but also imposes a significant burden on society. Estimates suggest that health care expenditures for individuals with depression are 50% to 100% higher than for those without depression [7]. Thus, effectively alleviating depression among older adults has become an urgent global health issue.

In recent years, information and communication technology has been recognized as a potential factor for alleviating depression in older adults [8]. Studies in early-aging countries such as the United Kingdom, the United States, Japan, and South Korea have confirmed that digital interventions can effectively alleviate depression among older adults [9-11]. Internet usage in Southeast Asia has experienced a significant upward trend in recent years. Statista reports that the internet penetration rate in the region was about 67.1% in 2024, with most countries exceeding a 70% penetration rate. However, smaller countries like Laos, Myanmar, and Timor-Leste have lower internet access rates, although improvements are ongoing. Digital technologies are increasingly applied in health management and social connectivity, with the widespread use of smartphones and wearable devices offering older adults comprehensive platforms for health monitoring and social interaction [12].

As the world’s largest high-income country, China had 119 million older internet users by the end of 2021, with an internet penetration rate of 43.2% among older adults. This raises a critical question: can internet usage effectively alleviate depression among older adults in China, and what are the potential mechanisms at play? Unfortunately, the academic community has paid insufficient attention to this issue, with few studies providing rigorous theoretical analysis and empirical evidence. To fill this gap, this paper investigates the causal relationship between internet usage and depression among older adults from both theoretical and empirical perspectives. The study aims to elucidate the underlying mechanisms and assess the impact of internet usage, thereby providing evidence from high-income countries to improve the mental health of older adults.

The primary challenge of this study is the inherent issue of reverse causality between internet usage and depression among older adults. Internet usage may influence depression among older adults, and conversely, depression may affect internet usage among older adults. This potential endogeneity problem significantly disrupts causal inference, greatly reducing the reliability of the regression coefficients. Existing studies have inadequately addressed this issue. The 2-stage instrumental variable (IV) method is widely regarded as one of the most effective approaches to overcome potential endogeneity issues [13]. By using an exogenous instrument, this method aims to eliminate the endogeneity of the primary variable, thereby enabling precise estimation of the regression coefficients. This approach requires the IV to be correlated with the primary variable while being exogenous to the error term.

This paper innovatively uses the National Big Data Comprehensive Pilot Zone policy as an IV for internet usage, using the 2-stage IV method to achieve more accurate causal identification. The National Big Data Comprehensive Pilot Zone policy, through the development of age-friendly applications and digital literacy training, significantly increases internet usage among older adults, meeting the relevant criterion. Simultaneously, depression among older adults does not influence the National Big Data Comprehensive Pilot Zone policy, satisfying the exogeneity criterion. In addition, to further eliminate endogeneity caused by omitted variables, this paper controls both time-fixed effects and individual-fixed effects in the 2-stage IV approach.

Compared with existing studies, this paper offers several potential contributions: First, this study provides a rigorous analysis of the causal relationship between internet usage and depression among older adults, both theoretically and empirically, thereby contributing to the literature on the mental health of older adults. Second, it is the first to use the 2-stage IV approach for a stringent empirical examination, effectively mitigating endogeneity issues in regression analysis, and thereby enriching existing empirical methodologies. Third, this paper conducts a heterogeneity analysis across different regions, expanding on current research that mostly focuses on heterogeneity based on demographic characteristics, thus broadening the scope of existing studies. Finally, we offer several specific and actionable policy recommendations, providing a basis for decision-making to improve the mental health of older adults in other high-income countries.

The remainder of this paper is organized as follows: (1) section 2 proposes testable theoretical hypotheses; (2) section 3 discusses empirical design, including data sources, econometric models, and variable definitions; (3) section 4 presents a series of empirical tests and provides an in-depth discussion of the results; and (4) section 5 concludes with the findings, policy recommendations, and limitations.

### Theoretical Hypotheses

#### Social Interaction Effect

Social networks refer to stable relationships formed through mutual connections among individuals and social members, encompassing both online and offline networks. Among these, family and friend networks constitute the most crucial offline social networks for older adults. However, due to physical decline, generation gaps, and children moving away or being busy with work, older adults find it challenging to maintain offline social networks. With the proliferation of the digital society, the internet provides a new social platform for older adults, helping them engage in social interactions, learning, and shopping, thereby enhancing their connection with the outside world. As of June 2023, China had approximately 140 million internet users aged 60 years and older, and this proportion continues to expand with increasing internet penetration. Internet usage not only alleviates loneliness among older adults but also expands their social relationships, prevents social isolation, and increases interaction frequency with family and friends [14].

Enhanced social networks have a significantly positive impact on the psychological well-being of older adults. Social interactions through the internet reduce feelings of loneliness and depression among older adults [15]. Social engagement enhances their sense of achievement, happiness, and security, serving as a crucial pillar for their integration into society and promotion of psychological health. The activity theory suggests that older adults can achieve self-awareness, bridge the gap with society, lessen loneliness, and attain higher life satisfaction through new social engagements. Increasing participation in both conventional and novel social activities is a valuable objective for improving the mental health of the increasingly aging population. The Social Convoy Theory also highlights the protective role of social relationship networks on psychological health. Therefore, by strengthening family and friend networks, the internet alleviates loneliness among older adults, promotes psychological health, and effectively reduces depression, a phenomenon termed here as the social interaction effect. Based on this, hypothesis 1 is proposed.

#### Hypothesis 1—Internet Usage Mitigates Depression Among Older Adults Through the Social Interaction Effect

##### Physical Exercise Effect

A series of studies have shown that internet usage among older adults transcends temporal and geographical constraints, enhances interaction with sports service providers, and offers high-quality, convenient, and diverse sports services [16,17]. In the era of big data, the internet also accurately delivers nearby sports information and suitable sports equipment, thus lowering barriers to the participation of older adults in physical activities. Older adults who frequently use the internet engage in physical exercise and moderate- to high-intensity activities more frequently each week. This suggests that the internet significantly boosts older adults’ enthusiasm for physical activity by providing convenience, scientific guidance, interactivity, and entertainment.

Increasing physical exercise has a significant positive impact on the psychological health of older adults. Physical exercise can enhance the cardiovascular function and physical fitness of older adults, promote health, delay aging, reduce the incidence of age-related diseases, and improve sleep quality. Research indicates that physical activity enhances subjective well-being [18], effectively treats depression, and reduces anxiety [19]. Based on the active aging theory and health demand model, studies have found that regular physical exercise significantly increases the likelihood of social participation among older adults [20]. Therefore, through increasing physical exercise, older adults not only improve their physical health but also significantly alleviate depression and enhance overall quality of life. We refer to this mechanism as the “physical exercise effect.” Based on this, hypothesis 2 is proposed.

#### Hypothesis 2—Internet Usage Alleviates Depression Among Older Adults Through the Physical Exercise Effect

##### Intergenerational Contact Effect

The current high population mobility in China often leaves adult children unable to care for their older parents due to work or study commitments, resulting in a shift in social roles and interpersonal relationships for older adults upon retirement. This situation limits their access to information and deepens their sense of social isolation, impacting their psychological and mental health [21]. The development of internet technology has provided convenient means for older individuals to stay in touch with their children, overcoming temporal and spatial barriers. This has significantly increased the frequency and quality of interactions between older parents and their children, thereby enhancing intergenerational support and subsequently improving the older adults’ subjective well-being [22].

According to the social support buffering model and main effects model, intergenerational support plays a crucial role in alleviating depression among older adults. On the one hand, intergenerational support helps mitigate the impact of resource loss and buffers the effects of stress on the depression of older adults. On the other hand, intergenerational support provides older adults with positive social evaluations for effective parenting, directly reducing their depression [23]. The cognitive behavioral theory of depression emphasizes that cognitive assessments are crucial variables influencing depression [24]. Intergenerational support meets the primary social goal of emotional management for older adults and fosters a more positive attitude in cognitive assessments, effectively reducing depression among older adults. We refer to this mechanism as the “intergenerational contact effect” and propose hypothesis 3 based on this premise.

#### Hypothesis 3—Internet Usage Mitigates Depression Among Older Adults Through the Intergenerational Contact Effect

##### Educational Enhancement Effect

With the widespread adoption of the internet, an increasing number of older individuals are engaging in online education, learning new knowledge and skills through virtual courses. This allows them to find new joys and values in the learning process [25,26]. The internet enables older individuals to access a broader range of information and knowledge, enhancing cognitive abilities and better adapting to social changes, thereby reducing feelings of loneliness caused by information isolation. In summary, the internet provides new learning opportunities for older individuals, expands their knowledge boundaries, and profoundly impacts their education.

Education can effectively mitigate the negative impact of age, gender, and family structure on the psychological health of older individuals [27-29]. As individuals age, their physical fitness gradually declines, but higher levels of education can help older individuals enhance their objective awareness of their health status, thereby influencing their psychological well-being. Studies indicate a positive correlation between cognitive function and life satisfaction among older individuals [30]. Education enhances the self-awareness and self-care abilities of older individuals, reducing psychological issues, and providing social and recreational opportunities, thereby improving mental health and enhancing overall quality of life. We refer to this mechanism as the educational enhancement effect. Based on this, hypothesis 4 is proposed.

#### Hypothesis 4—Internet Usage Alleviates Depression Among Older Individuals Through the Educational Enhancement Effect

**Figure 1:**
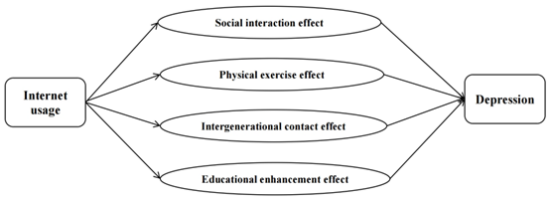
Conceptual framework.

Figure 1 below presents the conceptual framework of this study.

Internet usage serves as the primary exposure variable in this study, while depression is the main outcome variable. In addition, 4 potential mechanisms are explored: the social interaction effect, the physical exercise effect, the intergenerational contact effect, and the educational enhancement effect. These mechanisms are examined to better understand how internet usage might influence depression through various indirect pathways.

## Methods

### Data Sources

The data for this study primarily originate from the China Health and Retirement Longitudinal Study (CHARLS). CHARLS is a comprehensive, large-scale interdisciplinary survey led by the National School of Development at Peking University, conducted in partnership with the China Social Science Survey Center and the Youth League Committee of Peking University. The survey’s objective is to gather household and individual-level data on Chinese adults aged 45 years and older. CHARLS uses a probability proportional to size sampling method, systematically selecting samples from counties, villages, residential areas, households, and individuals. The national baseline survey was conducted in 2011, encompassing 150 counties (districts), 450 villages (residential areas), 10,257 households, and 17,708 individuals, offering a comprehensive representation of China’s middle-aged and older population. The CHARLS questionnaire collects data on demographics, family structure, employment and retirement, health status, and health-related behaviors. This study uses longitudinal panel data from 5 waves of CHARLS, spanning 2011 to 2020. The longitudinal data structure facilitates the analysis of individual-level changes over time, offering a more robust framework for examining causal relationships than cross-sectional data. In contrast to cross-sectional data, which captures a snapshot of individuals at a single point in time, longitudinal data enable the observation of changes within the same individuals across multiple time periods. This approach enhances our capacity to control unobserved individual heterogeneity and to more effectively assess temporal dynamics and causal mechanisms. Currently, CHARLS is recognized as the most authoritative and largest representative database for studying the health of older adults in China.

The data cleaning process proceeded as follows: (1) the data from the 5 survey rounds were matched and merged, resulting in a total of 96,616 observations; (2) missing values for the key explanatory and dependent variables were removed, totaling 15,735 (ie, 96,616–80,881); (3) further removal of missing values for control variables amounted to 1179 (ie, 80,881–79,702); and (4) subsequently, 2826 (ie, 79,702–76,876) singleton group values were removed. This resulted in a final sample of 76,876 observations for use in regression analysis.

### Econometric Equation

This study uses the IV regression method to address potential endogeneity issues in the data. Endogeneity occurs when an explanatory variable is correlated with the error term, often due to omitted variables, measurement errors, or reverse causality. This endogeneity can result in biased and inconsistent estimates of causal relationships. IV regression addresses this issue by using IVs that are correlated with the endogenous regressor but uncorrelated with the error term, providing a source of exogenous variation to identify causal effects. This method is particularly valuable in panel data analysis, where variable relationships are dynamic and prone to simultaneity bias. Using IVs allows for more reliable and unbiased estimates of causal effects, making this method well-suited for the study design. Thus, the IV approach offers a robust statistical framework to address endogeneity concerns and ensure the validity of causal inferences. The specific econometric model is as follows:

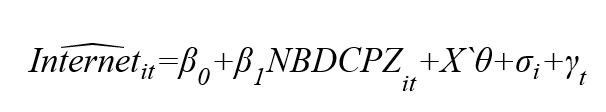

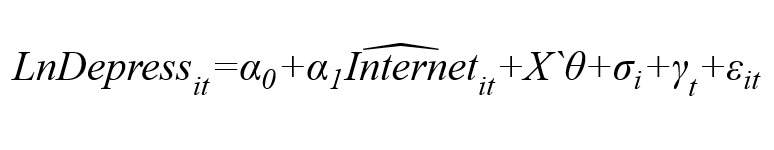





Equation (1) represents the first-stage regression, where 
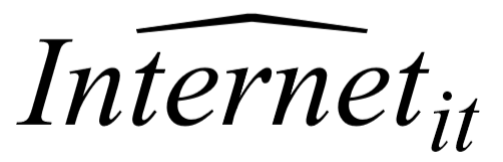
 denotes the fitted values of internet usage, *NBDCPZ_it_* represents the dummy variable for National Big Data Comprehensive Pilot Zones (NBDCPZ), *X`θ* denotes a series of control variables, *σ_i_* represents individual fixed effects to control for individual differences, and *γ_t_* represents time fixed effects to control for unobserved shocks across different years. Controlling for both time- and individual fixed effects aims to further eliminate endogeneity issues caused by omitted variables. The essence of the first-stage regression lies in regressing the endogenous variable *Internet_it_* on exogenous variables to obtain the fitted values 
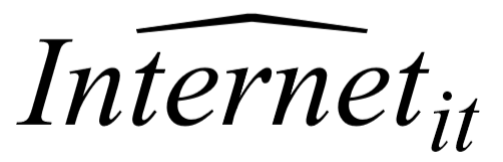
. Equation (2) represents the second-stage regression, where denotes depression among the older adults, *ε_it_* represents the error term, with other variables as in Equation (1). The essence of the second-stage regression lies in regressing *Lndepress_it_* on the instrumented 
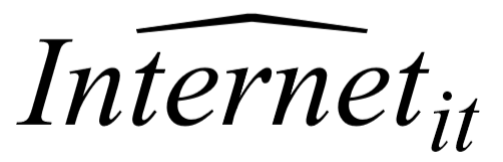
 to obtain accurate estimates of the coefficient *α_1_*.

### Variable Definition

#### Depression (LnDepress)

The level of depression among older adults was assessed using the widely recognized 10-item Center for Epidemiologic Studies Depression Scale (CESD-10). This scale consists of 10 items, each rated on a 4-point Likert scale ranging from 1 to 4, with items 5 and 8 reverse scored. The total score, derived by summing the individual item scores, reflects the level of depression, with higher scores indicating a greater risk of depression. To mitigate the impact of extreme values and facilitate the interpretation of results, the total depression score was logarithmically transformed.

#### Internet Usage (Internet)

Internet usage was assessed by asking respondents in the CHARLS survey, “Have you used the internet in the past month?” Responses were coded as 1 for “yes” and 0 for “no.” This core explanatory variable exhibits significant potential endogeneity issues with depression; higher levels of depression among older adults may inversely affect their internet usage behavior, potentially compromising the credibility of regression results.

#### Instrumental Variable (National Big Data Comprehensive Pilot Zone)

On March 1, 2016, China’s first NBDCPZ (Guizhou) received official approval from the National Development and Reform Commission, the Ministry of Industry and Information Technology, and the Cyberspace Administration of China. This pilot zone aims to conduct systematic trials in areas such as data resource management and sharing, data center integration, and data resource application. The plan is to develop a series of advanced big data products and cultivate key big data enterprises over 3 to 5 years, thereby promoting economic transformation and upgrading.

On October 8, 2016, these authorities issued another notice approving the establishment of additional NBDCPZ in 7 regions, including Beijing-Tianjin-Hebei. These newly approved national-level pilot zones comprise 2 interregional comprehensive pilot zones (Beijing-Tianjin-Hebei and the Pearl River Delta), 4 regional demonstration pilot zones (Shanghai, Henan, Chongqing, and Shenyang), and 1 pilot zone focused on the coordinated development of big data infrastructure (Inner Mongolia). The construction of these zones will facilitate trials and exploration in areas such as big data policy innovation, public data openness and sharing, big data innovation applications, big data industry clustering, big data element circulation, data center integration and use, and international cooperation in big data, thereby driving the innovative development of big data in China.

In this study, we creatively use the NBDCPZ as an IV for internet usage for older adults. This approach effectively addresses potential endogeneity issues, thereby enhancing the credibility of the regression results.

#### Control Variables

The selected control variables include gender, age, household registration, marital status, education, self-rated health, medical insurance, personal income, and alcohol consumption.

#### Mechanism Variables

In line with the theoretical hypotheses, the mechanism variables encompass the social interaction effect, physical exercise effect, intergenerational contact effect, and educational enhancement effect. Detailed measurement methodologies for these variables are provided in the section “Mechanism Explorations.”

### Descriptive Statistics

Table 1 presents the main variables and their descriptive statistics are as follows: (1) the mean score for depression (LnDepress) is 2.847 (SD 0.322); (2) the mean for internet usage (internet) is 0.149 (SD 0.356), indicating that 14.9% of the sample has used the internet; and (3) and the mean for NBDCPZ is 0.1 (SD 0.3), suggesting that 10% of observations are from the NBDCPZ. Details for other variables are omitted for brevity. It should be noted that in Table 1, the number of observations for variables is higher than in the regression tables. This discrepancy is due to the removalx of some singleton groups during the regression process. This deletion was automatic and did not substantively affect the conclusions of this study.

**Table 1:** Definition and description of main variables.

Variables	Observations, n	Mean (SD)	Definition
LnDepress	79,702	2.847 (0.332)	Using the CESD-10^a^ scale, scores were summed and logged
Internet	79,702	0.149 (0.356)	Respondents were asked whether they used the internet in the past month, with responses coded as 1 for “yes” and 0 for “no”
National Big Data Comprehensive Pilot Zone	79,702	0.1 (0.3)	If located in a pilot area, assign a value of 1 to years after the pilot period; otherwise, assign a value of 0
Gender	79,702	1.521 (0.5)	Male=1, female=2
Age	79,702	60.296 (9.731)	Current year: year of birth. Due to the presence of outliers, perform a 1% truncation of the upper and lower ends
Household registration	79,702	0.45 (0.497)	Rural household registration=1, others=0
Marital status	79,702	0.88 (0.325)	Married=1, others=0
Education	79,702	2.398 (4.116)	Not completed primary school=0, primary school=6, junior high school=9, senior high school or technical school=12, junior college=15, bachelor’s degree=16, master’s degree=19, doctorate=22
Self-rated health	79,702	2.78 (0.933)	Very poor=1, poor=2, fair=3, good=4, very good=5, excellent=6
Medical insurance	79,702	0.95 (0.219)	With medical insurance=1, other=0
Personal income	79,702	0.231 (0.421)	Received wages or bonuses in the past year=1, other=0
Alcohol consumption	79,702	2.375 (0.88)	In the past month, drank alcohol more than once=1, less than once=2, did not drink= 3
Social interaction effect	41,934	0.357 (0.455)	The frequency of social interaction activities was used as a measure, as detailed in the section “Mechanism Explorations”
Physical exercise effect	47,768	2.277 (0.552)	The frequency of physical exercise activities was measured as detailed in the section “Mechanism Explorations”
Intergenerational contact effect	34,020	1.871 (0.909)	The frequency of contact with children was used as a measure, as detailed in the section “Mechanism Explorations”
Educational enhancement effect	32,108	0.013 (0.115)	Measured by participation in school training courses over the past month, as detailed in the section “Mechanism Explorations”

^a^CESD-10: Center for Epidemiologic Studies Depression Scale.

### Ethical Considerations

The original data used in this study are derived from the CHARLS, which has been reviewed and approved by the Biomedical Ethics Review Committee of Peking University (approval IRB 00001052–11015). The current analysis is a secondary analysis of deidentified and publicly accessible data. This study does not involve any direct interaction with human participants or observation of individuals. In addition, this study does not involve any procedures or activities that may raise ethical concerns and complies with all applicable laws and regulations regarding research conduct. Therefore, no ethical approval is required.

## Results

### Benchmark Regression

To present comparative results, columns 1 and 2 in Table 2 show the results of ordinary least squares (OLS) regressions: column 1 indicates that the coefficient of internet usage (internet) on depression (LnDepress) is –0.05, significant at the 1% level. Column 2 presents the regression results further controlling for both individual fixed effects (FE) and year FE; the coefficient of internet usage “Internet” is –0.02, still significant at the 1% level. In Table 2, columns 3 and 4 present the results of the 2-Stage IV method: column 3 shows that the coefficient of internet usage “Internet” on depression (LnDepress) is –0.00, significant at the 1% level, with Kleibergen-Paap rk LM statistic and Cragg-Donald Wald F statistic tests confirming the validity of the instruments. Column 4 presents the regression results further controlling for individual FE and year FE; the coefficient of internet usage “Internet” is –0.01, and the instruments remain valid.

**Table 2:** Benchmark regression. Robust standard errors are in parentheses.

Variables	LnDepress			
	OLS^a^ (1)	OLS-FE^b^ (2)	IV^c^ (3)	IV-FE^d^ (4)
Internet	–0.0483^e^ (0.00308)	–0.0151^e^ (0.00373)	–0.00160^e^ (0.000209)	–0.0141^e^ (0.00350)
Gender	0.0745^e^ (0.00256)	0.00492 (0.0244)	0.000754^e^ (2.59e–05)	0.000501 (0.000398)
Age	0.000898^e^ (0.000126)	0.00380 (0.00426)	3.61e–06^f^ (1.53e–06)	–0.000119 (8.97e–05)
Household registration	0.0803^e^ (0.00228)	0.00178 (0.00369)	0.000779^e^ (2.36e–05)	0.000114 (8.08e–05)
Marital status	–0.0780^e^ (0.00369)	–0.0595^e^ (0.00742)	–0.000775^e^ (3.71e–05)	0.000146 (0.000225)
Education	–0.00563^e^ (0.000272)	–0.00101^e^ (0.000322)	–5.66e–05^e^ (2.75e–06)	–7.91e–05^e^ (1.85e–05)
Self-rated health	–0.0897^e^ (0.00123)	–0.0257^e^ (0.00122)	–0.000867^e^ (1.35e–05)	–0.000271^e^ (2.35e–05)
Medical insurance	–0.0126^f^ (0.00507)	–0.00371 (0.00490)	–8.55e–05^g^ (5.15e–05)	0.000173^g^ (0.000101)
Personal income	–0.0470^e^ (0.00277)	–0.0178^e^ (0.00317)	–0.000336^e^ (3.87e–05)	6.13e–05 (8.69e–05)
Alcohol consumption	0.00788^e^ (0.00140)	0.00465^f^ (0.00198)	5.29e–05^e^ (1.50e–05)	–0.000224^e^ (7.79e–05)
KP-LM^h^	No	No	1073.066	20.724
CD-Wald^i^	No	No	2000.060	26.327
Year fixed effect^j^	No	Yes	No	Yes
Individual fixed effect^k^	No	Yes	No	Yes
Robust^l^	Yes	Yes	Yes	Yes
Observations	79,702	79,702	79,702	76,876
*R^2^*	0.127	0.023	No	No

^a^OLS: ordinary least squares.

^b^OLS-FE: ordinary least squares-fixed effects.

^c^IV: instrumental variable.

^d^IV-FE: instrumental variable-fixed effects.

^e^*P*<.01.

^f^*P*<.05.

^g^*P*<.1.

^h^KP-LM: Kleibergen-Paap rk LM statistic, testing the relevance of IV.

^i^CD-Wald: Cragg-Donald Wald F statistic, testing the weak relevance of IV.

^j^Year FE: time fixed effects, controlling for unobserved shocks across different years.

^k^Individual FE: individual fixed effects, controlling for heterogeneity across individuals.

^l^Robust: the use of robust SEs, addressing heteroskedasticity effects.

The baseline regression results indicate the following: first, the coefficient significantly decreases after using IV regression, suggesting that the OLS regression overestimated the impact of internet usage on depression. Second, column 4, which simultaneously controls both individual FEs and year FEs, further mitigates endogeneity issues due to omitted variables. Therefore, column 4 is used as the baseline regression in this study, showing that internet usage significantly reduces depression scores by 1.41%. This finding indicates a significant alleviation of depression among older adults, consistent with the theoretical hypothesis.

Column 4 also reports the impacts of other control variables: education significantly reduces LnDepress, indicating that higher education levels ease depression. Self-rated health significantly reduces LnDepress, suggesting that better self-rated health decreases the likelihood of depression. Medical insurance significantly promotes LnDepress, implying that medical insurance does not alleviate depression. Alcohol consumption significantly reduces LnDepress, indicating that alcohol consumption is beneficial in alleviating depression. The other variables gender, age, household registration, marital status, and personal income do not show significant effects in the baseline regression. It is important to note that the reported *R*^2^ in the IV regression process has no practical significance; hence, it is not reported in this study [31].

### Robustness Checks

To examine the robustness of the baseline regression results, this section conducts a series of robustness tests, as detailed in Table 3: column 1 transforms the depression variable into a binary outcome, following the approach of Li and Luo (2024) [32], where depression scores exceeding 10 are assigned a value of 1, and 0 otherwise. Using the IV method based on a Probit model, the results indicate a significant reduction in depression due to internet usage (the coefficient is –2.116 and statistically significant at the 1% level), confirming robustness. Column 2 re-estimates the regression by internet usage frequency instead of binary internet usage. Frequency categories (almost daily=3, almost every week=2, not regularly=1) are derived directly from the CHARLS questionnaire. The results show a significant decrease in depression by 2.01%, reaffirming robustness. Column 3 replaces the IV method with propensity score matching to address potential selection bias in internet usage, where the choice of using the internet may be influenced by other factors, leading to biased regression coefficients. Using a 1:1 nearest-neighbor matching, the results indicate a significant reduction in depression by 0.0761%, demonstrating robust results. Column 4 uses a smart city pilot virtual variable as a new IV for the re-estimation. The results show a significant reduction in depression by 1.35%, confirming robustness. In addition, we conducted other robustness tests, such as re-estimating using the total depression score and applying a negative binomial regression model. The results remained robust across these specifications, and further details are omitted for brevity. In addition, we conducted weighted regressions at the individual, gender, household registration, household, community, and city levels. The results remained robust and are detailed in Multimedia Appendix 1.

**Table 3:** Robustness regression. Robust SE in parentheses.

	LnDepress			
Variables	Probit model (1)	Frequency (2)	Propensity score matching (3)	Smart city (4)
Internet	–2.116^a^ (0.53)	–0.0201^a^ (0.00111)	–0.000761^a^ (0.0000877)	–0.0135^a^ (0.00520)
Controls^b^	Yes	Yes	Yes	Yes
KP-LM^c^	No	182.559	No	6.757
CD-Wald^d^	No	304.661	No	5.604
Year FE^e^	Yes	Yes	Yes	Yes
Individual FE^f^	Yes	Yes	No	Yes
Robust^g^	Yes	Yes	Yes	Yes
Observations	88,359	2691	79,702	76,876
*R* ^2^	No	No	0.348	No

^a^*P*<.01.

^b^Controls represent a series of control variables.

^c^KP-LM: Kleibergen-Paap rk LM statistic, testing the relevance of IV.

^d^CD-Wald: Cragg-Donald Wald F statistic, testing the weak relevance of IV.

^e^Year FE: time fixed effects, controlling for unobserved shocks across different years.

^f^Individual FE: individual fixed effects, controlling for heterogeneity across individuals.

^g^Robust: the use of robust SEs, addressing heteroskedasticity effects.

### Mechanism Explorations

The empirical results above clearly demonstrate that internet usage significantly reduces depression levels among older adults. Moreover, the theoretical hypotheses proposed above have anticipated potential mechanisms. This section will therefore conduct mechanism tests to validate these theoretical hypotheses. Detailed results are provided in Table 4 below.

**Table 4:** Mechanism test. Robust standard errors are in parentheses.

Variables	Social interaction effect (1)	Physical exercise effect (2)	Intergenerational contact effect (3)	Educational enhancement effect (4)
Internet	0.492^a^ (0.244)	0.680^a^ (0.291)	0.374^a^ (0.151)	2.183^b^ (0.260)
Controls^c^	Yes	Yes	Yes	Yes
KP-LM^d^	6.943	22.750	9.698	No
CD-Wald^e^	8.832	24.261	10.196	No
Year FE^f^	Yes	Yes	Yes	Yes
Individual FE^g^	Yes	Yes	Yes	No
Robust^h^	Yes	Yes	Yes	Yes
Observations	38,860	47,905	30,362	38,985

^a^*P*<.05.

^b^*P*<.01.

^c^Controls represent a series of control variables.

^d^KP-LM: Kleibergen-Paap rk LM statistic, testing the relevance of IV.

^e^CD-Wald: Cragg-Donald Wald F statistic, testing the weak relevance of IV.

^f^Year FE: time fixed effects, controlling for unobserved shocks across different years.

^g^Individual FE: individual fixed effects, controlling for heterogeneity across individuals.

^f^Robust: the use of robust standard errors, addressing heteroskedasticity effects.

#### Social Interaction Effect

Regarding social interactions, the following activities were quantified: visiting friends in the past week; playing mahjong, chess, cards, or visiting community activity rooms; providing assistance to non-cohabitating relatives, friends, or neighbors; engaging in activities such as dancing, fitness, or qigong with clubs or organizations; volunteering or participating in charitable activities; and caring for non-cohabitating patients or disabled individuals. The total frequency of these activities was aggregated and log-transformed in this study. Column 1 indicates that internet usage increases the frequency of older social interactions by 49.2%, confirming the social interaction effect.

#### Physical Exercise Effect

Regarding physical exercise, the following activities were quantified: the number of days per week engaging in vigorous, moderate, or light physical activities for more than 10 minutes. The total frequency of these activities was aggregated and log-transformed in this study. Column 2 indicates that internet usage increases the frequency of older adults’ physical exercise by 68%, confirming the physical exercise effect.

#### Intergenerational Contact Effect

Regarding intergenerational contact, activities include contacting all children through phone, message, WeChat, mail, or email, and assigning reverse scores based on the frequency of these contacts as follows: almost every day=10, 2-3 times a week=9, once a week=8, every 2 weeks=7, once a month=6, once every 3 months=5, once every 6 months=4, once a year=3, almost never=2, other=1. The total scores of these frequencies were aggregated and log-transformed in this study. Column 3 indicates that internet usage increases the intergenerational contact scores of older adults by 37.4%, confirming the intergenerational contact effect.

#### Educational Enhancement Effect

Regarding educational enhancement, the following activity was quantified: whether the individual attended school or training courses in the past month. Clearly, this is a binary variable and should be regressed using the 2-stage IV method based on the Probit model. Column 4 demonstrates that internet usage significantly increases the likelihood of older adults receiving education, confirming the educational enhancement effect.

### Heterogeneity Analyses

To further enrich the understanding of the relationship between internet usage and depression, this study conducted a series of heterogeneity analyses, as shown in Table 5.

**Table 5:** Heterogeneity regression. Robust SEs are in parentheses.

	LnDepress					
Variables	Urban (1)	Village (2)	Eastern (3)	Noneastern (4)	Pilot (5)	Nonpilot (6)
Internet	–0.0174^a^ (0.00296)	–0.00774^a^ (0.00220)	–0.0185^a^ (0.00542)	0.00196 (0.00267)	–0.0190^b^ (0.0102)	–0.000707 (0.00393)
Controls^c^	Yes	Yes	Yes	Yes	Yes	Yes
KP-LM^d^	9.438	21.456	13.549	10.199	3.786	4.446
CD-Wald^e^	10.549	39.755	17.314	12.886	3.903	6.327
Year FE^f^	Yes	Yes	Yes	Yes	Yes	Yes
Individual FE^g^	Yes	Yes	No	Yes	Yes	Yes
Robust^h^	Yes	Yes	Yes	Yes	Yes	Yes
Observations	20,514	30,083	23,822	53,054	14,082	53,613

^a^*P*<.01.

^b^*P*<.1.

^c^Controls represent a series of control variables.

^d^KP-LM: Kleibergen-Paap rk LM statistic, testing the relevance of IV.

^e^CD-Wald: Cragg-Donald Wald F statistic, testing the weak relevance of IV.

^f^Year FE: time fixed effects, controlling for unobserved shocks across different years.

^g^Individual FE: individual fixed effects, controlling for heterogeneity across individuals.

^h^Robust: the use of robust SEs, addressing heteroskedasticity effects.

#### Comparison of Urban and Rural Areas

Urbanization exacerbates the hollowing-out problem in rural areas, leading to higher levels of loneliness and more severe depression among rural older adults [33]. The cumulative disadvantage hypothesis suggests that this urban-rural depression disparity stems from the urban-rural divide, where rural residents accumulate disadvantages that peak in old age. The diffusion of the internet has varying effects on alleviating depression among older adults in urban and rural areas. Due to better infrastructure, urban older adults benefit more from internet access, facilitating easier social participation, information access, and entertainment, thereby effectively reducing feelings of loneliness and depression [34]. Moreover, proximity to children’s residences and stable family support systems in urban areas, coupled with internet usage, further alleviates their depression. In contrast, rural older adults face limited internet access due to inadequate infrastructure, resulting in fewer opportunities to use internet benefits for social interaction and support. Combined with a lack of effective family support systems and communication channels, the internet’s impact in alleviating depression among rural older adults is less pronounced compared with their urban counterparts. Therefore, it is expected that the alleviating effect of internet usage on depression would be more significant in urban areas. The grouped regressions in columns 1 and 2 confirm this hypothesis, indicating that the alleviating effect of internet usage is higher in urban areas (–1.74%) compared with rural areas (–0.774%).

#### Comparison of Eastern and Noneastern Regions

In eastern China, older adults have more opportunities to engage in social activities, access health information, and participate in online entertainment through the internet, resulting in a more pronounced alleviation of depression [35]. Furthermore, the higher economic development in eastern regions generally correlates with higher levels of education and economic capability among older adults, facilitating easier access to and utilization of the internet for psychological well-being [36,37]. In contrast, the older adults in noneastern regions experience lower internet usage rates and breadth due to limited internet resources, technological barriers, and economic constraints, leading to less significant alleviation of depression compared with their counterparts in eastern regions [38]. Therefore, it is anticipated that the therapeutic effect of internet usage on depression in older adults is more pronounced in eastern China and remains limited in noneastern regions. According to the classification standards of the National Bureau of Statistics, eastern China includes the provinces (and cities) of Beijing, Tianjin, Hebei, Shanghai, Jiangsu, Zhejiang, Fujian, Shandong, Guangdong, and Hainan. The grouped regressions in columns 3 and 4 confirm this hypothesis, indicating that the alleviating effect of internet usage is higher in eastern regions (–1.85%) compared with noneastern regions (0.00196, which is statistically insignificant).

#### Comparison of Broadband China Pilot and Nonpilot Areas

Since its implementation in 2013, the “Broadband China” strategy has significantly enhanced broadband network coverage and accessibility through advanced network technologies and infrastructure development. In pilot areas, older adults have more frequently used the internet for social interactions and access to health information, resulting in a notable reduction in depression. In contrast, nonpilot areas, constrained by limited internet resources and technical support, offer fewer opportunities for internet usage by older adults, leading to less significant alleviation of depression compared with pilot areas [39]. Moreover, the widespread availability of broadband networks has facilitated increased older adults’ participation in social activities and access to more health services, further mitigating depression [40]. Therefore, this study anticipates that the therapeutic effect of internet usage is greater in the pilot areas of Broadband China and smaller in the nonpilot areas. The grouped regressions in columns 5 and 6 confirm this hypothesis, indicating that the alleviating effect of internet usage is higher in pilot areas (–1.9%) compared with nonpilot areas (–0.000707, which is statistically insignificant).

## Discussion

This study’s core finding is that internet usage significantly alleviates depression symptoms among older adults, with a reduction of 1.41%. This result suggests that internet usage alleviates depression through four main mechanisms: the social interaction effect, physical exercise effect, intergenerational contact effect, and educational enhancement effect. Specifically, internet usage facilitates social activities among older adults, helping to reduce loneliness and, in turn, alleviating depressive symptoms. In addition, the internet enables older individuals to more easily participate in physical activities, which improves both their physical and mental health. Furthermore, internet usage strengthens communication with children, enhancing intergenerational support and reducing depressive feelings. Finally, internet usage provides older individuals with access to more educational resources, which improves self-awareness and self-management, thereby reducing depression symptoms. In summary, this study provides empirical evidence on how internet usage alleviates depression in older adults through multiple pathways, highlighting the potential of the internet to improve mental health in older adults.

The findings of this study align with existing literature emphasizing the positive impact of internet usage on mental health in older adults. Internet usage reduces depression by alleviating social isolation [41] and enhancing intergenerational relationships [11]. In addition, it reduces loneliness through increased social contact [9,42] and improves psychosocial functions, including physical activity and education [43,44]. Our study expands on this by incorporating the mediating effects of physical activity and education in depression reduction. While excessive internet usage may lead to social media fatigue and worsen mental health [45], our findings suggest that appropriate internet usage fosters mental well-being, as indicated by Lam et al [8]. Furthermore, mobile health apps have been shown to reduce depression in other populations, supporting the efficacy of digital interventions [46]. Despite concerns about internet addiction [47], our results suggest that regulated internet usage can improve both mental and functional health, particularly in older adults [48]. By using a 2-stage IV approach, this study mitigates endogeneity issues, providing robust causal inferences consistent with previous research [49].

The study’s findings suggest several policy measures to enhance the mental health of older adults via internet usage, based on the identified mechanisms and observed heterogeneity in the effects of digital engagement: (1) strengthen internet infrastructure in rural and underserved regions: while internet usage enhances mental health through social interaction, physical activity, and educational access, its impact is greater in urban areas. Policy makers should prioritize the expansion of internet infrastructure in rural and noneastern regions to ensure that older adults can fully benefit from digital engagement. (2) Initiate targeted digital literacy programs: considering the influence of digital literacy on the effectiveness of internet usage, targeted training programs for older adults, particularly in rural areas, should be prioritized. These initiatives should emphasize teaching older users to engage in online social activities, access health resources, and participate in educational content, which have proven effective in reducing depression. (3) Create age-appropriate digital mental health platforms: mental health platforms should be customized for different subgroups. In urban areas, platforms should prioritize enhancing social interaction and intergenerational communication, whereas, in rural areas, the emphasis should be on bridging the digital divide by providing simple and accessible health and social resources. (4) Promote the development of age-friendly digital applications: policymakers should facilitate the creation of digital tools that encourage physical activity and cognitive stimulation among older adults. These applications can address mechanisms identified in the study, such as exercise and education, and contribute to reducing depression among older users.

While this study provides valuable insights into the relationship between internet usage and depression among older adults, several limitations must be considered. First, the reliance on self-reported data for depression and internet usage may introduce biases, such as social desirability or recall bias. Future studies could incorporate objective measures, such as log data from internet providers or wearable devices, to improve accuracy. Second, although the 2-stage IV approach addresses endogeneity, the exogeneity of the chosen instrument, the NBDCPZ, may still be questioned, and alternative instruments or experimental designs, like randomized controlled trials, could strengthen causal inference. In addition, the heterogeneity of internet usage effects across subgroups (eg, by age, education, or income) was not fully explored and warrants further investigation. Finally, while this study focused on short-term effects, future research should examine the long-term impacts of internet usage on the mental health of older adults, including potential risks such as internet addiction or over-reliance on digital platforms.

## Appendix

Multimedia Appendix 1 Weighted regression. Robust standard errors in parentheses.

## Abbreviations

CESD-10: Center for Epidemiologic Studies Depression Scale
CHARLS: China Health and Retirement Longitudinal Study
FE: fixed effects
IV: instrumental variable
NBDCPZ: National Big Data Comprehensive Pilot Zone
OLS: ordinary least squares

## Data Availability

The datasets generated during this study are available from the corresponding author on reasonable request.
