# Supplementary material for: Impact of Internet Usage on Depression Among Older Adults: Comprehensive Study
Source: J Med Internet Res. 2025 Jan 31;27:e65399. doi: 10.2196/65399 (PMC11829179; doi:10.2196/65399)
Supplement: Multimedia Appendix 1 [file jmir_v27i1e65399_app1.docx]

**Table S1.**

|  | LnDepress | | | | | |
| --- | --- | --- | --- | --- | --- | --- |
| Variables | Individual-level weighting | Sex-based weighting | Hukou-based weighting | Household-level weighting | Community-level weighting | City-based weighting |
|  | (1) | (2) | (3) | (4) | (5) | (6) |
| Internet | -0.0147*** | -0.00810*** | -0.0125** | -0.0158*** | -0.0159*** | -0.0135*** |
|  | (0.00439) | (0.00288) | (0.00502) | (0.00487) | (0.00490) | (0.00373) |
| Controls^a^ | Y | Y | Y | Y | Y | Y |
| KP-LM^b^ | 14.123 | 14.443 | 7.998 | 12.833 | 12.741 | 17.097 |
| CD-Wald^c^ | 24.075 | 18.356 | 12.789 | 21.572 | 21.386 | 29.645 |
| Year FE^d^ | Y | Y | Y | Y | Y | Y |
| Individual FE^e^ | Y | Y | Y | Y | Y | Y |
| Robust^f^ | Y | Y | Y | Y | Y | Y |
| Observations | 76,876 | 36,717 | 30,620 | 76,876 | 76,876 | 76,876 |
| ****P*<.01.  ***P*<.05.  **P*<.1.  ^a^Controls represent a series of control variables.  ^b^KP-LM: represents Kleibergen-Paap rk LM statistic, testing the relevance of IV.  ^c^CD-Wald: represents Cragg-Donald Wald F statistic, testing the weak relevance of IV.  ^d^Year FE: denotes time fixed effects, controlling for unobserved shocks across different years.  ^e^Individual FE: denotes individual fixed effects, controlling for heterogeneity across individuals.  ^f^Robust: indicates the use of robust standard errors, addressing heteroskedasticity effects. | | | | | | |
